# Critical values and variation in type I error along chromosomes in the COGA dataset using the applied pseudo-trait method

**DOI:** 10.1186/1471-2156-6-S1-S54

**Published:** 2005-12-30

**Authors:** George J Papanicolaou, Cristina M Justice, Illija M Kovac, Alexa JM Sorant, Alexander F Wilson

**Affiliations:** 1 Genometrics Section, Inherited Disease Research Branch, NHGRI/NIH, Baltimore, MD, USA

## Abstract

**Background:**

By analyzing a "pseudo-trait," a trait not linked or associated with any of the markers tested, the distribution of the test statistic under the null hypothesis can provide the critical value for the appropriate percentile of the distribution. In addition, the anecdotal observation that *p*-values tend to be more significant near the telomeres was investigated.

**Results:**

The applied pseudo-trait (APT) method was applied to the Affymetrix and Illumina SNPs in the Collaborative Study on the Genetics of Alcoholism dataset to determine appropriate critical values for regression of offspring on mid-parent (ROMP) and Haseman-Elston association and linkage analyses, investigating the occurrence of type I errors in different chromosomal locations, and the extent to which the critical values obtained depend on the type of pseudo-trait used.

**Conclusion:**

On average, the 5 percentile critical values obtained for this study were less than the expected 0.05. The distribution of *p*-values does not seem to depend on chromosomal position for ROMP association analysis methods, but does in some cases for Haseman-Elston linkage analysis. Results vary with different pseudo-traits.

## Background

The vast majority of markers in a genomic screen for linkage or association are not linked or associated with the trait being analyzed. This observation led to the applied pseudo-trait (APT) method [1,2], a method devised to determine empirically critical values appropriate for a particular study (including family structure, marker map, type of analysis, genotyping errors, etc.). To determine this critical value, the distribution of the test statistic under the null hypothesis is created by analyzing a "pseudo-trait" which is not linked to or associated with any of the markers with which it is tested. Any marker with a known chromosomal location can be used to generate a "pseudo-trait" and tested with non-syntenic markers. Another option is to use an unrelated random deviate, which can be used with all markers. The appropriate percentile of the distribution of the resulting test statistics can be taken to be the critical value. While similar in spirit to permutation and simulation methods, APT incorporates the actual pedigree and marker values and requires less computational time. The type of trait used as a pseudo-trait may effect the distribution of the null hypothesis. However, the extent to which this occurs is not known.

This method can also be used to investigate so-called "spurious" peaks that occur at the ends of chromosomes. A number of anecdotal accounts by our group and others suggest that values near the telomeres tend to be inflated [Rice J, personal communication; Atwood L, personal communication]. This observation has been noted in both two-point and multipoint linkage analysis. In this study we used APT to determine the distribution of the type I errors across chromosomes.

Three objectives were addressed: 1) to determine the appropriate critical values for the Affymetrix and Illumina single-nucleotide polymorphism (SNP) datasets through the use of the APT method for linkage analysis (with the revised Haseman-Elston method) and association analysis (with variations of the regression of offspring on mid-parent (ROMP) method); 2) to determine the validity of the observation that the type I error rate may vary with respect to chromosomal location; 3) to determine to what extent the critical values generated is dependent on the type of pseudo-trait used.

## Methods

Several different pseudo-traits were considered for use with the Collaborative Study on the Genetics on Alcoholism (COGA) dataset. For each chromosomal arm, a SNP was randomly chosen from a region excluding centromeric (SNPs 10 MB proximal to the centromere) [3] and telomeric DNA (SNPs at the ends of the provided genetic map). The selected SNP was required to have a minor allele frequency ≥ 0.10. The position of the SNP was confirmed using public databases when available. Suitable markers could not be obtained for the acrocentric (chromosome groups D and G) *p *arms of chromosomes 13–15 and 21–22, leaving 39 pseudo-trait markers. For each of these markers, an allele-count pseudo-trait was defined as the number of occurrences of allele 2 at that marker. Another type of pseudo-trait was created by adding a random quantity drawn from a standard normal distribution to the allele count. In addition, two randomly generated pseudo-traits were also created: one from a standard normal distribution and one from a uniform distribution. A total of 80 pseudo-traits were considered.

For every combination of pseudo-trait and SNP marker, linkage analysis was performed using the revised Haseman-Elston method with single-point identity-by-descent (IBD) sharing probabilities and using the mean-corrected cross-product as the dependent variable, as implemented in S.A.G.E. 4.5 [4]. Association analysis was carried out with variations of the ROMP method [5]. These variations included ROMP, requiring phenotype data on both parents, and a combination of ROMP and ROOP (Regression on One Parent), which uses trios with incomplete parental data [6]. The tests of association were performed both using one randomly selected member from each sibship (ROMP-one, ROMP/ROOP-one) and also using all sibs (ROMP-all, ROMP/ROOP-all). Although originally formulated as a test for parent-offspring trios, inclusion of (a few) additional offspring has been shown to have little effect on the properties of the test. All versions of ROMP were coded in R (version 1.4) [7].

*p*-Values resulting from all SNP tests were determined for each pseudo-trait. For pseudo-traits based on a SNP marker, tests with syntenic markers were excluded, so that null hypothesis conditions were maintained. For each chromosome, the set of *p*-values obtained was considered as a single unit and also broken down by location on the chromosome (*p term*, *q term*, and middle). Each chromosome end (*p term *and *q term*) was defined as 10% (or 25%) of the chromosome, according to physical map distance. Alternative definitions of chromosomal ends using a fixed distance of 3 MB (Affymetrix) and 7 MB (Affymetrix and Illumina) were also investigated. For some chromosome arms, too few SNPs (less than 0.5% of all markers) were available in the terminal segment, and those segments were omitted from the partitioned segment summaries. For each segment and for the whole genome, descriptive statistics were obtained using SAS version 8.2 [8].

To investigate the possible variation in type I error rate between telomeric and non-telomeric regions, means of each segment were computed separately. In addition, traditional analysis of variance and Kolmogorov-Smirnov two-sample tests were performed separately to compare each end with the middle segment (i.e., *p term *v. middle and *q term *v. middle).

Affymetrix and Illumina data were considered separately throughout the study.

## Results

Critical values corresponding to a 5% significance level for the COGA dataset were taken to be the 5^th ^percentile of the distribution of *p*-values from marker tests from the whole genome. For the Affymetrix data these values ranged for different pseudo-traits from 0.005–0.058, 0.010–0.063, 0.006–0.059, 0.001–0.065, 0.001–0.051, for Haseman-Elston, ROMP-one, ROMP/ROOP-one, ROMP-all, ROMP/ROOP-all analyses, respectively.

The distribution of *p*-values was also examined separately for different types of chromosomal segments to investigate possible differences in type I error rate corresponding to physical location. Distribution means are presented in Table 1 (decile) and Table 2 (7 MB), summarized over all pseudo-traits for the Affymetrix dataset. Direct observation suggests a slight increase in *p*-value at the *q *ends.

Analysis of variance and Kolmogorov-Smirnov tests (with all chromosomes pooled) comparing *q term *segment values with mid-chromosome segment values and those comparing *p term *segment values with mid-chromosome segment values showed non-significant results for most pseudo-traits for ROMP-based association analysis (data not shown). However, comparisons for Haseman-Elston linkage analysis did show significant results for some pseudo-traits. For example, using the decile segment definition, 41 of 80 pseudo-traits showed significant (at the 0.01 level) differences between the *q term *segment means and the middle segment means, according to both analysis of variance and Kolmogorov-Smirnov tests. *P*-values for different pseudo-traits ranged from 0–0.8, with a mean of 0.1.

Fifth percentile values for different types of pseudo-traits are summarized in Table 3 for the Affymetrix dataset. Although there is considerable variation among critical values generated using different pseudo-traits, there does not appear to be a systematic difference between types of pseudo-traits for Haseman-Elston analysis. However, with ROMP using all sibs (ROMP-all, ROMP/ROOP-all) there appears to be a discernable decrease in the generated critical value when a normal variate is involved.

Similar results were observed for the Illumina dataset.

## Discussion

The determination of critical values with the APT method resulted in a large range of values. Overall the fifth percentile critical values obtained were less than the expected 0.05 level. Although the fifth percentile of the whole genome *p*-value distribution was, on average, less than 1 standard deviation below 0.05 for three of the ROMP methods (ROMP-one, ROMP/ROOP-one, ROMP-all), the fifth percentiles for the ROMP/ROOP method using all the sibs and the revised Haseman-Elston method were less than expected. This suggests that these methods may be liberal when used with nominal *p*-values. Alternatively, one could use a critical value derived with the APT method, but the range of empiric critical values obtained in this fashion is large.

With respect to the differences between means of *p*-values across segments, there appeared to be little difference for the ROMP methods for tests of association. However, significant differences between the *q term *segment and the middle segment were seen for some pseudo-traits with the revised Haseman-Elston linkage analyses. This seems to corroborate the anecdotal reports for linkage analyses.

Finally, the type of pseudo-trait chosen may in some cases have an effect on the resulting null distribution. However, there may be a large variation among critical values generated using different pseudo-traits of any given type. Additional studies and simulations will be required to investigate the statistical properties of the estimate of the critical value.

## Abbreviations

APT: Applied pseudo-trait

COGA: Collaborative Study on the Genetics of Alcoholism

ROMP: Regression of offspring on mid-parent

ROOP: Regression on one parent

SNP: Single-nucleotide polymorphism

## Authors' contributions

All authors read and approved the final manuscript. Contributions: analysis (GJP, CMJ, and AJMS, with assistance by IMK), theory (GJP and AFW), overall concept (AFW), first draft, and project supervisor (GJP).

## Figures and Tables

**Table 1:** Mean of *p*-value distribution, summarized over all 80 pseudo-traits using decile definitions (Affymetrix data)

	Whole genome	*p term *deciles^b^	Mid-chromosome	*q term *deciles
Haseman-Elston				
mean	0.503	0.499	0.503	0.506
SD	0.018	0.032	0.021	0.032
No. SNPs^a^	9857–10803	842–969	7936–8691	993–1129

ROMP-one				
mean	0.496	0.496	0.496	0.498
SD	0.015	0.020	0.015	0.017
No. SNPs^a^	9817–10802	834–968	7896–8691	984–1129

ROMP/ROOP-one				
mean	0.487	0.487	0.487	0.488
SD	0.021	023	0.022	0.022
No. SNPs^a^	9769–10802	832–968	7861–8691	984–1129

ROMP-all				
mean	0.479	0.479	0.478	0.483
SD	0.032	0.033	0.032	0.031
No. SNPs^a^	9845–10807	839–969	7925–8695	989–1129

ROMP/ROOP-all				
mean	0.456	0.455	0.456	0.458
SD	0.034	0.036	0.034	0.033
No. #SNPs^a^	9825–10807	837–969	7910–8695	989–1129

^a^The number of SNPs varies due to missing data and variable numbers of markers excluded by synteny.

^b^The *p *ends of chromosomes 13, 14, 21, 22 could not be used.

**Table 2:** Mean of *p*-value distribution, summarized over all 80 pseudo-traits using 7 MB definition of chromosome ends (Affymetrix data)

	Whole genome	*p term *(7 MB)^b^	Mid-chromosome	*q term *deciles^c^
Haseman-Elston				
mean	0.503	0.502	0.503	0.506
SD	0.018	0.035	0.019	0.037
No. SNPs^a^	9857–10803	383–437	9059–9968	330–386

ROMP-one				
mean	0.496	0.494	0.496	0.498
SD	0.015	0.023	0.015	0.020
No. SNPs^a^	9817–10802	380–436	9023–9968	330–386

ROMP/ROOP-one				
mean	0.487	0.485	0.487	0.488
SD	0.021	0.028	0.021	0.026
No. SNPs^a^	9769–10802	380–436	9016–9968	330–386

ROMP-all				
mean	0.479	0.479	0.478	0.483
SD	0.032	0.035	0.032	0.034
No. SNPs^a^	9845–10807	381–437	9051–9972	330–386

ROMP/ROOP-all				
mean	0.456	0.453	0.456	0.459
SD	0.034	0.042	0.034	0.035
No. SNPs^a^	9825–10807	381–437	9030–9972	330–386

^a^The number of SNPs varies due to missing data and variable numbers of markers excluded by synteny.

^b^The *p *ends of chromosomes 13, 14, 15, 19, 21, 22 could not be used.

^c^The *q *ends of chromosomes 9, 16, 17 could not be used.

**Table 3:** 5th percentile of whole genome p-value distribution, by type of pseudo-trait (Affymetrix data)

	All pseudo-traits	Allele-count pseudo-traits	Normal + allele-count pseudo-traits	Uniform	Normal
# pseudo-traits	80	39	39	1	1

Haseman-Elston					
mean	0.010	0.011	0.007	0	0
SD	0.039	0.036	0.043	0.040	0.046

ROMP-one					
mean	0.009	0.008	0.010	0	0
SD	0.048	0.049	0.047	0.059	0.039

ROMP/ROOP-one					
mean	0.043	0.044	0.041	0.059	0.039
SD	0.011	0.011	0.012	0	0

ROMP-all					
mean	0.015	0.009	0.012	0	0
SD	0.038	0.049	0.028	0.042	0.029

ROMP/ROOP-all					
mean	0.013	0.012	0.013	0	0
SD	0.026	0.029	0.023	0.042	0.029
